# Molecular characterization and phylogeny of Shiga toxin-producing *Escherichia coli* derived from cattle farm

**DOI:** 10.3389/fmicb.2022.950065

**Published:** 2022-08-04

**Authors:** Shiqin Zhang, Zhiye Bai, Zichen Wang, Xiang Wang, Wen Wang, Hongmei Li, Qingli Dong

**Affiliations:** ^1^School of Health Science and Engineering, University of Shanghai for Science and Technology, Shanghai, China; ^2^State Key Laboratory for Managing Biotic and Chemical Threats to the Quality and Safety of Agro-Products, MOA Laboratory of Quality & Safety Risk Assessment for Agro-Products (Hangzhou), Institute of Quality and Standard of Agricultural Products, Zhejiang Academy of Agricultural Sciences, Hangzhou, China

**Keywords:** STEC, food, cgMLST, serogroups, phylogenetic relationships

## Abstract

Shiga toxin-producing *Escherichia coli* (STEC) is an important food-borne pathogen, which can cause diseases such as diarrhea, hemorrhagic enteritis, and hemolytic uremic syndrome in humans. Twelve STEC isolates were collected from beeves and feces of commercial animals in China between 2019 and 2020 for this study. In addition to the determination of serotype and Shiga toxin subtype, whole-genome sequencing (WGS) was used for determining phylogenetic relationships, antimicrobial resistance (AMR), virulence genes, and sequence type (ST) of isolates. A total of 27 AMR genes were detected, and each STEC isolate carried more than 10 AMR genes. Eight STEC isolates from ground beef and four STEC isolated from feces were screened. A total of seven serotypes were identified, and one isolate ONT:H10 was undetermined by SeroTypeFinder. Three O157:H7 strains were confirmed and the remaining five serogroups were confirmed as O26:H11, O81:H31, O105:H8, O178:H19, and O136:H12. The phylogenetic analysis showed that STEC isolates of the same serotype or ST were clustered together based on cgMLST. The comparison of the genomes of 157 STEC reference isolates worldwide with our local STEC isolates showed that STEC isolates screened in China represented various collections and could not form a separate cluster but were interspersed among the STEC reference collection, which suggested that several STEC isolates shared a common ancestor irrespective of STEC serotype isolates. cgMLST revealed that isolates of the same O serotype clustered irrespective of their H type. Further investigation is required to determine the pathogenic potential of other serotypes of STEC, particularly in regard to these rare serotypes.

## Introduction

Shiga toxin-producing *Escherichia coli* (STEC) is an important food-borne pathogen causing zoonotic diseases. As a subpopulation of diarrheagenic *E. coli* (DEC), STEC can result in severe cases of disease such as hemorrhagic colitis (HC) and hemolytic uremic syndrome (HUS). The pathogenicity of STEC is associated with the production of Shiga-like toxins (*Stx*) encoding either one or both of the *stx*_1_ and *stx*_2_ genes (Gonzalez and Cerqueira, 2020). Since STEC was first described in 1982, more than 400 different serotypes of STEC have been identified, and research hotspots have focused on O157:H7 (Karmali et al., 2003). Moreover, several non-O157 serotypes have been associated with sporadic cases and outbreaks, such as O26, O45, O103, O111, O121, and O145, which were considered as the top six non-STEC serogroups (Mathusa et al., 2010; Amézquita-López et al., 2018).

In China, the outbreak of STEC was traced back in Xuzhou, Jiangsu province in 1999, which led to 195 hospitalized HUS patients and 177 deaths (Wang et al., 2011). The traceability analysis revealed that the food was contaminated by the fecal shedding of courtyard animals carrying STEC O157:H7 (Xiong et al., 2012). Although the main reservoir of STEC was considered to be cattle, there has been an increase in non-bovine food-related outbreaks worldwide (Smith et al., 2014). Some researches show that the STEC was transmitted mainly through foods, such as raw poultry, fresh vegetables, fruits, water, ground beef, and dairy products, to humans (Smith et al., 2014). Various transmission routes of STEC have been proposed, and the primary one is presumably *via* consumption of contaminated food or water (Parsons et al., 2016). However, some transmissions are not clear. For epidemiologic purposes, several genetic fingerprinting methods have been used to classify, trace, and prevent the dissemination of STEC (Heir et al., 2000). Pulsed-field gel electrophoresis (PFGE) and multilocus variable-number of tandem-repeat (MLVA) have mostly been applied in analyzing the molecular epidemiology of STEC and proved to be reliable among the sequence-based methods. However, low discriminatory power is exposed in the methods of complex workflows, expensive reagents, and time-consuming process.

The advances in next-generation sequencing in the past decade have made it true to perform WGS of organisms, including STEC at affordable costs (Joensen et al., 2014, 2015), and there was an ever-increasing evidence demonstrating that WGS was not only epidemiologic typing to detect and support outbreak investigations but also to define transmission pathways of pathogens and to reveal laboratory cross-contamination (Keoser et al., 2012; Chattaway et al., 2016; Baha et al., 2019). WGS data have the potential to provide powerful, high-level phylogenetic analysis and to show insight into the evolutionary background of the outbreak strains by using quantifiable genetic differences (Baltasar et al., 2014; Mikhail et al., 2018). For example, the comparison of 62 STEC local isolates from Chile with STEC isolated from the rest of the world by core genome multilocus sequence typing (cgMLST) typing method showed Chile STEC did not cluster with genomes of the rest of the world, suggesting local STEC isolates and STEC isolated from worldwide were not phylogenetical (Smith et al., 2014; Gutierrez et al., 2021) and indicating STEC phylogeny was affected by the origin of geographical isolation.

An earlier study described the diversity of Chinese *E. coli* O157 obtained from different sources such as mutton, beef, chicken, pork, and vegetable salad. Twenty-two pulsotypes by PFGE and 23 types by MLVA were found, and this study demonstrated the diversity among 30 STEC O157 isolates (Li et al., 2015). However, other STEC serotypes have not been studied in China. In this study, non-O157 STEC and O157 STEC were mainly isolated from cattle feces and ground beef in China, and serotype, sequence type (ST), and virulence genes of these strains were characterized. Furthermore, based on WGS data, cgMLST analysis from STEC isolates screened by our lab and National Center for Biotechnology Information (NCBI) confirmed phylogenetic relationships among different STEC serotypes found and suggested possibly high-risk foods causing STEC. The comparison of these characteristics with those of foodborne and clinical isolates around the worldwide could provide some information for food safety risk assessment.

## Materials and methods

### Collection of STEC strains

Between 2019 and 2020, the STEC isolates used in this study were isolated from animal food and feces in Shanghai. All samples were collected in sterile sample bags and transported in ice as soon as possible to the laboratory for immediate processing. The enrichment method was modified from the GB4789.6-2016 food microbiological examination of *Escherichia coli* (National Food Safety Standards of China). Briefly, 225 ml of sterile Tryptone Soya Broth was added to a sterile sample bag with 25 g of each sample. Then, incubated at 37°C for 16–22 h on a shaking platform (200 rpm). Enrichment solutions were inoculated into CHROMagar™ STEC plates (CHEOMagar, Pairs, France). Discrete, strongly mauve colonies were picked and streaked out on MacConkey agar (MAC) and sorbitol MacConkey agar (CT-SMAC; Hopebio, Qingdao, China) for 18–22 h (John et al., 2018). STEC was identified by using PCR for the targeted genes *wzxO157, stx*_1_, and *stx*_2_ screening, as shown in Table 1. All positive samples were further processed to obtain pure *stx*-positive isolates (Boer et al., 2015).

**Table 1:** Sequences of all primers and annealing temperature used in this work.

**Primers**	**Direction**	**Sequence (5′-3′)**	**Product size (base)**	**Annealing temperature (**°**C)**	**Reference**
*stx_1_*	Forward	AAATCGCCATTCGTTGACTACTTCT	370	58	GB4789.6-2016
	Reverse	TGCCATTCTGGCAACTCGCGATGCA			
*stx_2_*	Forward	CAGTCGTCACTCACTGGTTTCATCA	283	58	
	Reverse	GGATATTCTCCCCACTCTGACACC			
*wzxO157*	Forward	CGGACATCCATGTGATATTGG	259	28	
	Reverse	TTGCCTCTGTACAGCTAATCC			

### DNA extraction

Genomic DNA of STEC isolates was extracted from overnight cultures using boiling. Briefly, 0.5 g of feces and 25 g of food samples were added together into 225 ml of lysogeny broth (LB) and incubated at 37°C for 18–24 h. The enrichment broth (1 ml) was centrifuged at 4,000 rpm for 2 min, then centrifuged at 12,000 rpm for 5 min, and supernatant was removed. Finally, 200-μl sterile deionized water was added and boiled at 100°C for 15 min.

### Whole-genome sequencing

Whole-genome sequencing (WGS) using a 400-bp paired end was performed on the STEC genomic DNA on an Illumina Novaseq according to the manufacturer's instructions. The genomic DNA libraries were prepared using the TIANamp Bacteria DNA Kit (Tiangen Biotech Beijing Co., Ltd., China). The raw data were assembled using SPAdesv3.11.1 software to obtain scaffolds sequences with default parameters, and raw data were cleaned by Fastp (v0.19.4; number of bases to average across: 4, average quality required: 20, fold coverage was required to be >30 for the cleaned data) (Bolger et al., 2014; Shifu et al., 2018).

### Data analysis and molecular characterization

The serotype of each STEC isolate was determined by uploading the assembled genomes to the SerotypeFinder 2.0 (https://cge.cbs.dtu.dk/services/SerotypeFinder/) of the Center for Genomic Epidemiology (CGE) website: the threshold of identity was set to 85% and the minimum length was set to 60%, VirulenceFinder 2.0 web-based tool (https://cge.cbs.dtu.dk/services/VirulenceFinder/) was used to determine virulence genes of each STEC isolates in this study with the following parameters: the 90% threshold identity and 60% minimum length. ResFinder 4.1 web-based tool (https://cge.food.dtu.dk/services/ResFinder/)was used to determine AMR genes for each STEC isolate with default parameters. The sequence types (STs) were identified by uploading assembled genomes to the MLST Finder (https://cge.cbs.dtu.dk/services/MLST/) (Jaureguy et al., 2008).

### Phylogenetic analysis of STEC isolates based on cgMLST

To determine the phylogenetic relationship of the isolates, a gene-by-gene approach was performed by SeqSphere (v3.1.1-rc04, Ridom) and BLAST (v2.2.12) (Michaela et al., 2021). The key parameters identity was 90% and aligned was 100%. Briefly, a core genome MLST scheme was developed using the genome of *E. coli* O157:H7 strain Sakai (accession no. NC_002695; https://www.cgmlst.org/ncs/schema/8896773/) as a reference genome and an additional eleven *E. coli* as query genomes to extract open reading frames (ORFs) from the genome of each isolate using MLST+ (v2.11.0+) of SeqSphere (v3.1.1-rc04, Ridom). The genes shared by all isolates analyzed were defined as the core genome for phylogenetic analysis. Loci were detected by chewBBACA (https://github.com/B-UMMI/chewBBACA), BLAST Score Ratio threshold was 0.8, and the number of loci present in genomes was 95% (Jagadesan et al., 2019). By default, a minimum spanning tree (MST) was calculated based on loci, which were carried out using SeqSphere (v3.1.1-rc04, Ridom).

The phylogenetic relationship of the STEC isolates of this study with isolates of the STEC reference collection (*n* = 157; Supplementary File 1) from NCBI was determined by cgMLST method. The download criteria for raw data of reference collection were as follows: (1) sequence depth cannot be lower than 200×, and (2) sequence length in the range of 4.7–5.5 M.

## Results and discussion

### STEC detection and isolation

As shown in Table 2, 289 samples from cattle feces, lettuce, and ground beef were tested during 2019–2020, 4.41% (9/204) of cattle feces, 1.81% (1/55) of lettuce, and 0.00% (0/30) of ground beef were *stx*_1_ and *stx*_2_-positive. Further, 4 STEC strains were isolated from nine STEC-positive samples of cattle feces, whereas no STEC strains were found in any samples of lettuce that tested positive for STEC. The prevalence of STEC in cattle feces was higher than the other samples, with a PCR-positive rate of 4.41% and an isolation rate of 1.96%. Eight STEC strains were isolated from ground beef earlier, and 12 STEC strains were collected for this study. STEC has emerged as an important food-borne pathogen, several reports indicated that STEC cases have been transmitted to humans *via* food (Smith et al., 2014; Jenkins et al., 2019; Mohammad et al., 2021). In this study, STEC isolates were collected from cattle feces and ground beef in Shanghai, China. The findings in this study supported that cattle carry various serotypes (Monaghan et al., 2012).

**Table 2:** Prevalence of STEC isolates in cattle feces, lettuce, and ground beef samples.

**Year**	**Method**	**Cattle feces (%)**	**Lettuce (%)**	**Ground beef (%)**	**Total (%)**
2019–2020	PCR^a^	9/204 (4.41%)	1/55 (1.81%)	0/30 (0.00%)	10/289 (3.46%)
	Culture^b^ confirmed	4/204 (1.96%)	0/55 (0.00%)	0/30 (0.00%)	4/289 (1.38%)

a Samples consists of stx_1_ or stx_2_ gene identified by multiplex PCR were considered to be positive.

b PCR positive samples were further cultured and at least one isolate was isolated by CHROMagar™ STEC agar.

### The analysis of molecular characterization

The serotypes of 12 STEC isolates were identified by Serotypefinder software. In addition to the common serotypes such as O157:H7 and O26:H11, other serotypes such as O81:H31, O136:H12, and O105:H8 were isolated. However, an unknown STEC serotype ONT:H10 was found, which may be due to the incomplete coverage of the area involved in O-antigen determination, resulting in the generation of unknown serotype STEC. As a result, three O157:H7 strains were confirmed and the remaining five serogroups were O26:H11, O81:H31, O105:H8, O178:H19, and O136:H12. In this study, seven housekeeping (*adk, fumC, gyrB, icd, mdh, purA*, and *recA*) genes were used for MLST analysis, and seven STs were identified, as shown in Table 3. Three O157:H7 were ST 11, four O136:H12 belong to ST 329. O26:H11 clustered in ST 21, ONT:H10 clustered in ST 441, O178:H19 clustered in ST 192, O81:H31 clustered in ST101, and O105:H8 clustered in ST 13.

**Table 3:** Sequence types and virulence genes detected by WGS in 12 STEC strains in this study.

	**MRL380001 Ground beef**	**MRL380002 Ground beef**	**MRL380003 Ground beef**	**MRL380004 Ground beef**	**MRL380005 Ground beef**	**MRL380006 Ground beef**	**MRL380007 Ground beef**	**MRL380008 Ground beef**	**MRL380009 Cattle feces**	**MRL380010 Cattle feces**	**MRL380011 Cattle feces**	**MRL380012 Cattle feces**
Serotype	O157:H7	O26:H11	O157:H7	O157:H7	ONT:H10	O81:H31	O178:H19	O105:H8	O136:H12	O136:H12	O136:H12	O136:H12
Sequence type	ST11	ST21	ST11	ST11	ST441	ST101	ST192	ST13	ST329	ST329	ST329	ST329
*astA*	**+**	**+**	**+**	**+**	**–**	**–**	**–**	**+**	**–**	**+**	**+**	**+**
*chuA*	**+**	**–**	**+**	**+**	**–**	**–**	**–**	**–**	**–**	**–**	**–**	**–**
*eae*	**+**	**+**	**+**	**+**	**–**	**–**	**–**	**–**	**–**	**–**	**–**	**–**
*ehxA*	**+**	**+**	**+**	**+**	**–**	**+**	**–**	**–**	**–**	**–**	**–**	**–**
*espF*	**+**	**+**	**+**	**+**	**–**	**–**	**–**	**–**	**–**	**–**	**–**	**–**
*espP*	**–**	**+**	**+**	**+**	**+**	**+**	**+**	**–**	**–**	**–**	**–**	**–**
*etpD*	**+**	**–**	**+**	**+**	**–**	**–**	**–**	**–**	**–**	**–**	**–**	**–**
*gad*	**+**	**+**	**+**	**+**	**+**	**+**	**+**	**+**	**+**	**+**	**+**	**+**
*hra*	**–**	**+**	**+**	**+**	**–**	**–**	**–**	**–**	**–**	**–**	**–**	**–**
*iss*	**+**	**+**	**+**	**+**	**–**	**–**	**+**	**+**	**+**	**+**	**+**	**+**
*nleA*	**+**	**+**	**+**	**+**	**–**	**–**	**–**	**–**	**–**	**–**	**–**	**–**
*nleB*	**+**	**+**	**+**	**+**	**–**	**–**	**–**	**–**	**–**	**–**	**–**	**–**
*nleC*	**+**	**+**	**+**	**+**	**–**	**–**	**–**	**–**	**–**	**–**	**–**	**–**
*ompT*	**+**	**+**	**+**	**+**	**–**	**–**	**+**	**–**	**+**	**+**	**+**	**+**
*stx_1_*	**+**	**+**	**+**	**+**	**–**	**–**	**–**	**+**	**+**	**+**	**+**	**+**
*stx_2_*	**+**	**+**	**+**	**+**	**+**	**+**	**+**	**–**	**–**	**–**	**–**	**–**
*terC*	**+**	**+**	**+**	**+**	**+**	**+**	**+**	**+**	**+**	**+**	**+**	**+**
*tir*	**+**	**–**	**+**	**+**	**–**	**–**	**–**	**–**	**–**	**–**	**–**	**–**
*traT*	**+**	**+**	**+**	**+**	**+**	**+**	**–**	**–**	**+**	**+**	**+**	**+**
*IpfA*	**–**	**–**	**–**	**–**	**–**	**–**	**–**	**–**	**+**	**–**	**+**	**–**

+, detected; –, not detected.

A total of 20 virulence genes were detected among the 12 STEC strains. The distribution of virulence genes among strains of different serogroups is shown in Table 3. Glutamate decarboxylase-encoding *gad* gene and tellurium ion resistance protein-encoding *terC* were the most widespread gene and were detected in all strains. Other well-known virulence genes found were as follows, with their distribution noted in parentheses: *eae* which encodes intimin (33.3%, *n* = 4), *astA* which encodes heat-stable enterotoxin (66.7%, *n* = 8), and *ompT* which encodes outer membrane protease (75%, *n* = 9). Four isolates with the combination of *stx*_1_ and *stx*_2_ as the most frequently detected type were found. Three isolates O157:H7 and one O26:H11 with *stx*_1_ and *stx*_2_ virulence genes were acquired, and the non-O157 serotype STEC isolated from ground beef with single *stx*_2_ was found. This result was consistent with previous studies in China, which reported that *stx*_1_ and *stx*_2_ were common in retail meat or slaughterhouses (Leung et al., 2001; Li et al., 2015, 2016). All the STEC obtained from 204 cattle feces were O136:H12 serotype and only contained *stx*_1_ virulence gene. WGS characterization of the isolates revealed that 12 isolates possessed AMR genes that can confer resistance to at least six classes of antimicrobials. As shown in Table 4, 27 AMR genes were detected, and each STEC isolate carried more than 10 AMR genes, suggesting that 12 STEC isolates may be multidrug-resistant. According to the health outcome of reported confirmed human STEC cases, serogroups O81:H31, ONT:H10 were classified to group E indicating non-human only disease, serogroups O178:H19, O105:H8 serotype were classified to group A/B/C indicating a HUS-associated serotype (Hazards, 2013). All those STEC isolates distributions indicated a higher diversity of serotypes in cattle and revealed a potential threat to consumers.

**Table 4:** Summary of resistance genes carried by STEC isolates.

**Description**	**AMR genes**
β-Lactam resistance genes	*ompC, mdrA, oppA, ampG, arcB, rtcB, mppA, arcA, ampH*
Vancomycin resistance genes	*ddl, murG, mraY, murF, alr, vanX*
Fluoroquinolones resistance genes	*emrB, MdtH, emrK, EvgA, MdtE, AcrE*
Nitroimidazole resistance genes	*MsbA*
Peptide resistance genes	*PmrF*
Aminoglycoside resistance genes	*BaeS, MdtB, tolC, cpxA*

### cgMLST scheme

The cgMLST scheme including 3,152 cgMLST targets, 1,485 accessory targets (Supplementary File 2), and 567 low-quality genes were filtered out. According to the cgMLST typing scheme, based on genome typing with PanGen.py in the chewBBACA tool, which uses Prodigal annotation, a total of 2,514 loci were selected as cgMLST targets shared by 12 STEC and 2,586 loci were selected as cgMLST targets shared by 169 STEC (Supplementary Files 3, 4). The MST was constructed based on the loci for the analysis of phylogenetic relationship with default.

### Phylogenetic comparison of STEC isolates and reference collections

**Figure 1:**
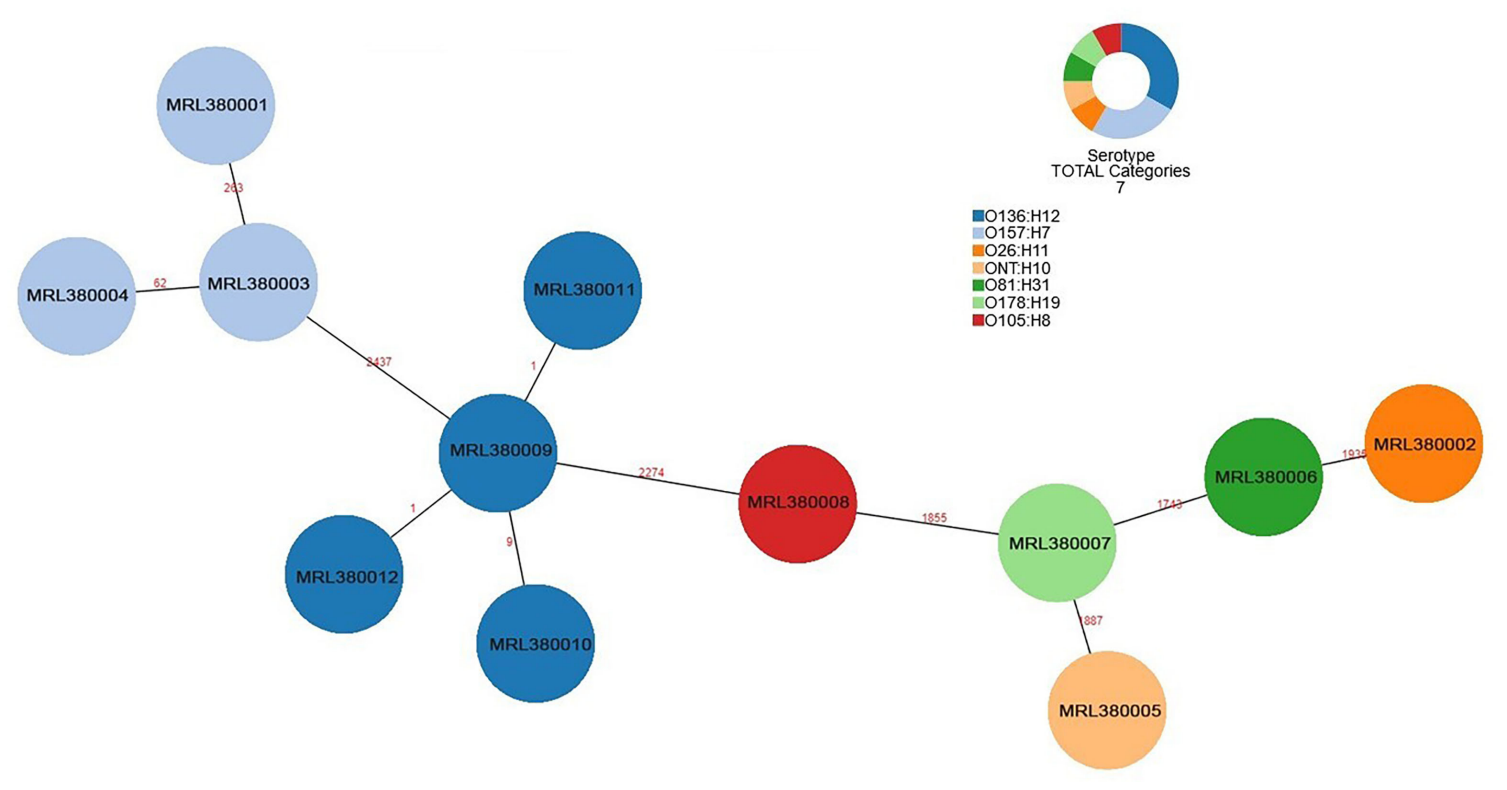
Minimum spanning tree for 12 STEC isolates from 289 samples based on the 2,514 loci, a total of 7 serotypes, different colors represent different serotypes.

**Figure 2:**
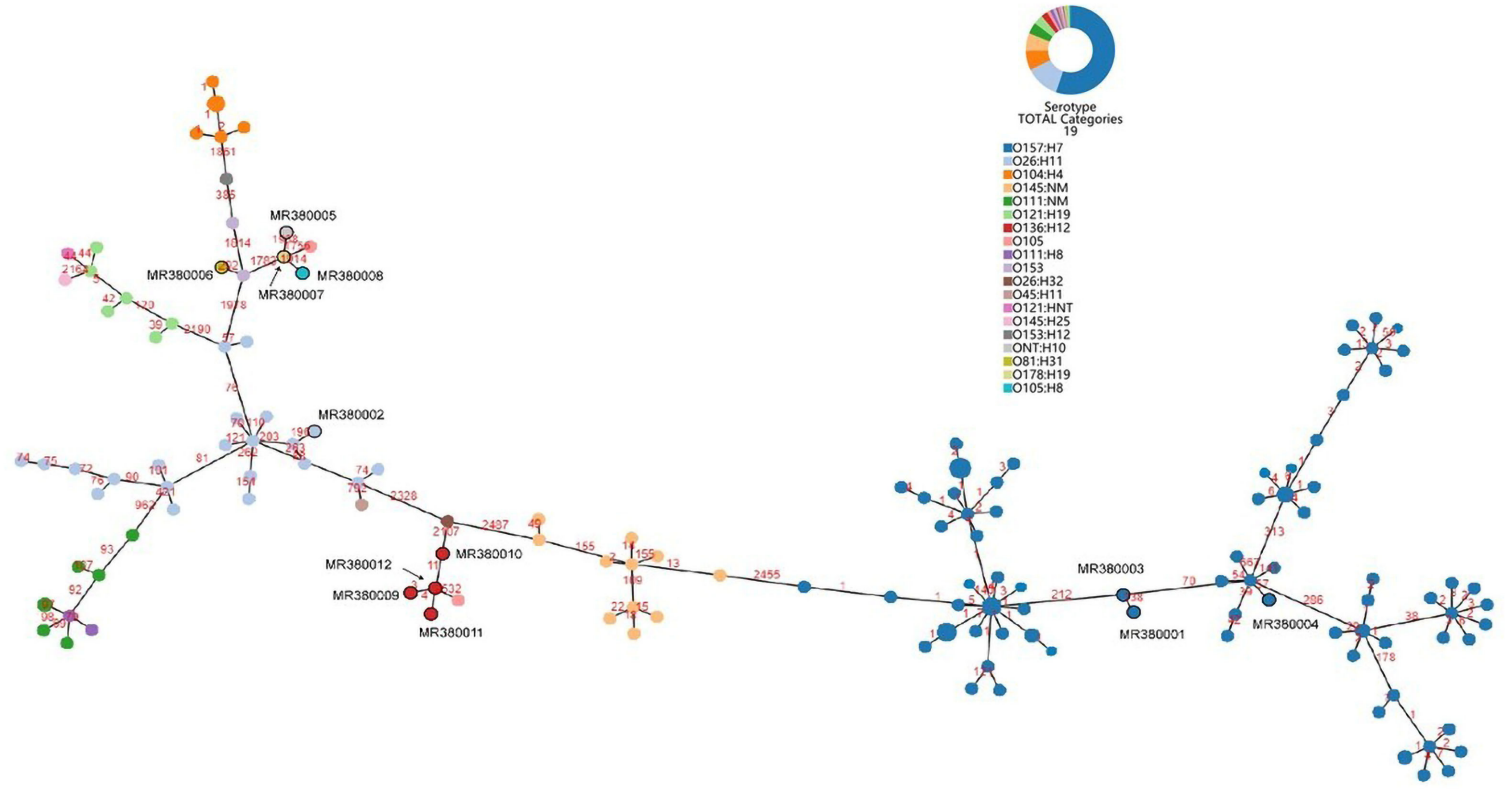
Minimum spanning tree for 12 STEC isolates from 289 samples and 157 STEC strains from NCBI based on the 2,586 loci, a total of 19 serotypes, different colors represent different serotypes.

The number of locus differences in core genome MLST minimum spanning tree ranged from 1 to 2,437 between 12 STEC isolates (Figure 1). Isolates of the same serotype are clustered together, four O136:H12 (MRL380009, MRL380010, MRL380011, and MRL380012) can be considered homologous because of their small allelic differences. Figure 2 shows a minimum spanning tree representing 12 STEC isolates from our lab and 157 STEC reference collections (Supplementary File 1) by cgMLST. In this study, STEC isolates representing various serotypes collections and without forming a separate cluster suggested that STEC isolates were phylogenetically related to STEC reference collections, on the one hand. On the other hand, epidemiologically related strains grouped together or were even part of a clonal cluster as shown for strains concurrently isolated from the farm. The top six non-STEC serotypes O145:H25 and four local isolated STEC strains O136:H12 clustered together, suggesting O136:H12 may be pathogenic. In order to determine the relatedness between different serotype STEC strains, the present study calculated the allelic distances between strains ranging from 1 to 2532. The greatest distances were observed between the O136:H12 and O145:H25. In addition, the same somatic antigen (O antigen) and the different flagella antigen (H antigen) STEC were divided into same lineages, such as O121:HNT, O121:H19 and O111:NM, O111:H8.

Based on sequencing data and *in silico* analysis, STEC isolates from our lab and STEC reference collection strains revealed that the STEC isolates represented a heterogeneous group. A minimum spanning tree was constructed by all 169 STEC strains based on cgMLST method illustrated that STEC of the same serotype or ST were clustered together. However, STEC strains of the same O serogroup were located in the same phylogenetic clusters, isolates of the same H type, irrespective of their O serotype may not share a common ancestor. This finding was not consistent with the results from cgMLST typing analysis revealing H serogroups were described as monophyletic, while O serogroups were described as polyphyletic (Ju et al., 2012; Steyert et al., 2012; Ferdous et al., 2016).

Several limitations existed in this study. Firstly, STEC isolation and identification methods should be further improved, because parasites in cow manure can cause false positives during the initial screening process (Ferdous et al., 2016). STEC strain false positives can be decreased but not entirely eliminated by choosing CHROMagar™ STEC plates, MacConkey agar, and sorbitol MacConkey agar. The limited local STEC isolates and lacking clinical isolates might have influenced the reported diversity.

## Conclusion

In conclusion, the results analyzing 12 isolates from food sources and feces of commercial animals, indicated a low prevalence of STEC in Shanghai, China. The capability of WGS providing rapid data for identification, serotyping, sequence typing, and virulence genes of STEC strains compared with traditional methods was further confirmed. STEC strains of the same O serogroup were located in the same phylogenetic clusters, isolates of the same H type, irrespective of their O serotype may not share a common ancestor by cgMLST. The study revealed that cgMLST typing method could be useful for outbreak investigations of STEC strains. In addition, the data stemmed from wide-ranging molecular characteristics with WGS resolution could be used as an effective approach for comparing with similar human, food, or animal isolates at the international level.

## Data availability statement

The data presented in the study are deposited in the Genome Sequence Archive (Genomics, Proteomics & Bioinformatics 2017) in National Genomics Data Center (Nucleic Acids Res 2021), accession number CRA007022.

## Author contributions

QD and HL conceived of the study and modified the first draft of the article. SZ is responsible for the experimental work, article writing, and data analysis. ZB and ZW isolated laboratory strains. XW and WW provided help with research ideas. All authors reviewed and approved the final manuscript.

## Funding

This study was supported by foundation 2010DS700124-KF2001 from State Key Laboratory for Managing Biotic and Chemical Threats to the Quality and Safety of Agro-products, China.

## Conflict of interest

The authors declare that the research was conducted in the absence of any commercial or financial relationships that could be construed as a potential conflict of interest.

## Publisher's note

All claims expressed in this article are solely those of the authors and do not necessarily represent those of their affiliated organizations, or those of the publisher, the editors and the reviewers. Any product that may be evaluated in this article, or claim that may be made by its manufacturer, is not guaranteed or endorsed by the publisher.
