# Supplementary figures and images for: Molecular characterization and phylogeny of Shiga toxin-producing Escherichia coli derived from cattle farm
Source: Front Microbiol. 2022 Aug 4;13:950065. doi: 10.3389/fmicb.2022.950065 (PMC9386476; doi:10.3389/fmicb.2022.950065)

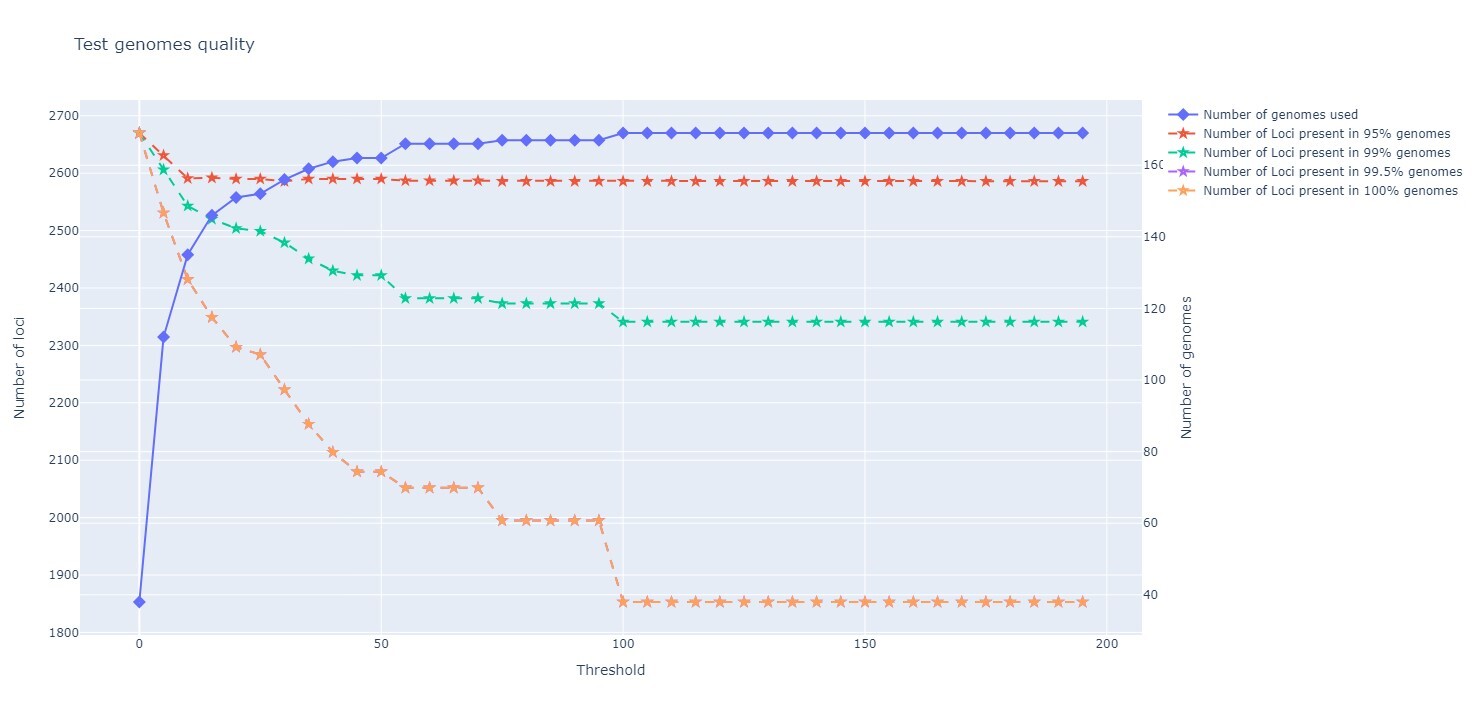

Supplement: Supplementary file 4 [file Image_1.JPEG]
